# Change in Alzheimer’s Disease Blood-Based Biomarkers and Associations With Cortical Thickness

**DOI:** 10.1093/geroni/igaf122.210

**Published:** 2025-12-31

**Authors:** James Pike, Yifei Lu, Keenan Walker, Thomas Mosley, Rebecca Gottesman, Clifford Jack, Josef Coresh, Priya Palta

**Affiliations:** New York University, New York, New York, United States; Merck, Landsdale, Pennsylvania, United States; National Institute on Aging, Baltimore, Maryland, United States; University of Mississippi Medical Center - The MIND Center, Jackson, Mississippi, United States; National Institutes of Health, Bethesda, Maryland, United States; Mayo Clinic, Rochester, Minnesota, United States; New York University, New York, New York, United States; University of North Carolina School of Medicine, Chapel Hill, North Carolina, United States

## Abstract

Blood-based biomarkers show promise as a noninvasive method for measuring Alzheimer’s disease pathology and neurodegeneration throughout the lifecourse. However, the relationship between longitudinal changes in these biomarkers and late-life brain morphology in community-dwelling populations requires further investigation. Between 2011 and 2013, 1,977 participants from the Atherosclerosis Risk in Communities Study received underwent 3 Tesla magnetic resonance imaging scans. Whole-brain cortical thickness was quantified by Freesurfer. Stored plasma samples collected in midlife (1993-95, mean age 59.0 years) and late-life (2011-13, mean age 76.8 years) from a subsample of 1,515 participants (60.7% female, 25.5% Black adults) were assayed in 2022 using Quanterix SiMoA. The assay quantified amyloid-β (Aβ)42/40, phosphorylated tau at threonine 181 (p-Tau181), neurofilament light (NfL), and glial fibrillary acidic protein (GFAP). Linear regression models estimated the association of blood-based biomarkers from midlife, late-life, and the change from midlife to late-life with cortical thickness in late-life. In models adjusted for demographics, lifestyle factors, cardiovascular factors, and the presence of APOE ε4 alleles, higher midlife measures of p-Tau181 and increases from midlife to late-life in NfL and GFAP were associated with lower cortical thickness in late-life. Concurrent measures of p-Tau181, NfL, and GFAP were associated with lower cortical thickness in late-life and exhibited a stronger association among participants ≥75 years old compared to participants <75 years old. Additional longitudinal research is needed to determine whether blood-based biomarkers predict cortical thinning over time and may serve as an early indicator of accelerated brain atrophy.

